# 340 Chorioamnionitis diagnosis and implications for cesarean delivery surgical site infection surveillance

**DOI:** 10.1017/ash.2026.10681

**Published:** 2026-06-23

**Authors:** Jiyun Ha, JaHyun Kang

**Affiliations:** 1 Seoul National University; 2 Seoul National University College of Nursing

## Abstract

**Background:** Airborne infection isolation rooms (AIIRs) are a highly restricted environment in which patients’ physical and emotional care needs are intensified. In AIIRs, nurses spend the most time in close contact with patients, rendering the nurse-patient relationship particularly important. However, empirical research exploring nurse-patient relationships among patients isolated for infectious diseases remains limited. The Fundamentals of care (FOC) framework conceptualizes patients' physical and psychosocial needs and the nursing activities required to address them, positioning the nurse-patient relationship as the core dimension through which care is integrated and delivered within specific care contexts. This study aimed to explore nurse–patient relationships as experienced by patients in AIIRs and to interpret these experiences from the perspective of the FOC framework. **Methods:** Participants were adults aged 18 years or older who had been hospitalized for infectious diseases in AIIRs for at least three days. To ensure voluntary participation and authenticity of responses, patients who were not directly cared for by the researcher were recruited. Data were collected from 11 patients residing in AIIRs at a tertiary general hospital through semi-structured, one-on-one in-depth interviews conducted between July 6 and August 31, 2025. Interviews were audio-recorded and transcribed. Data were analyzed using thematic analysis following Braun and Clarke, employing a hybrid approach that integrated deductive coding based on pre-established FOC concepts with inductive coding derived from participants’ narratives. **Results:** The nurse–patient relationship experienced by patients in AIIRs was characterized by an overarching theme, a relationship of striving together along a long journey until returning to the world. Five main themes, comprising twelve subthemes, were identified: (1) feeling safe enough to entrust oneself, (2) experiencing continuous and attentive care, (3) experiencing prepared care, (4) recognizing the nurse as the one who understands me best, and (5) a relationship built together. Analysis revealed that patients in AIIRs experienced positive nurse–patient relationships through the development of trust and nurses’ attentive responses to their needs, which facilitated a shift from isolation-related anxiety to emotional stability. Furthermore, relationship development was described as a mutual process, with both nurses and patients actively engaged. **Conclusion:** This study suggests that nurse-patient relationship formation grounded in the FOC framework within AIIRs contributes to patients’ psychological stability and perceived recovery. The findings indicate the need for FOC-based nursing education, structured communication practices, and consideration of the AIIRs environment and institutional policies to support relationship-centered care in isolation settings.

